# Microbial Ecology of Oxygen Minimum Zones Amidst Ocean Deoxygenation

**DOI:** 10.3389/fmicb.2021.748961

**Published:** 2021-10-27

**Authors:** Andrew M. Long, Sophie K. Jurgensen, Ariel R. Petchel, Emily R. Savoie, Jennifer R. Brum

**Affiliations:** Department of Oceanography and Coastal Sciences, Louisiana State University, Baton Rouge, LA, United States

**Keywords:** oxygen minimum zone (OMZ), viruses, nutrient cycles, microeukaryotes, microbial ecology, global climate change

## Abstract

Oxygen minimum zones (OMZs) have substantial effects on the global ecology and biogeochemical processes of marine microbes. However, the diversity and activity of OMZ microbes and their trophic interactions are only starting to be documented, especially in regard to the potential roles of viruses and protists. OMZs have expanded over the past 60 years and are predicted to expand due to anthropogenic climate change, furthering the need to understand these regions. This review summarizes the current knowledge of OMZ formation, the biotic and abiotic factors involved in OMZ expansion, and the microbial ecology of OMZs, emphasizing the importance of bacteria, archaea, viruses, and protists. We describe the recognized roles of OMZ microbes in carbon, nitrogen, and sulfur cycling, the potential of viruses in altering host metabolisms involved in these cycles, and the control of microbial populations by grazers and viruses. Further, we highlight the microbial community composition and roles of these organisms in oxic and anoxic depths within the water column and how these differences potentially inform how microbial communities will respond to deoxygenation. Additionally, the current literature on the alteration of microbial communities by other key climate change parameters such as temperature and pH are considered regarding how OMZ microbes might respond to these pressures. Finally, we discuss what knowledge gaps are present in understanding OMZ microbial communities and propose directions that will begin to close these gaps.

## Introduction

In addition to rising temperatures and ocean acidification, deoxygenation is one of the key effects of climate change on marine ecosystems. Predictions of future ocean deoxygenation are largely driven by rising water temperatures that decrease the solubility of O_2_ in water. Global ocean deoxygenation is predicted to further extend oxygen minimum zones (OMZs), whose expansion has been documented since the 1960s (Falkowski et al., 2011; Horak et al., 2016; Breitburg et al., 2018). Thus, the study of OMZs is vital to understanding how climate change may alter marine environments. Moreover, the current knowledge of OMZ formation, expansion, and the microbial ecology of OMZs may prove useful in predicting how marine environments will be altered upon deoxygenation.

**FIGURE 1:**
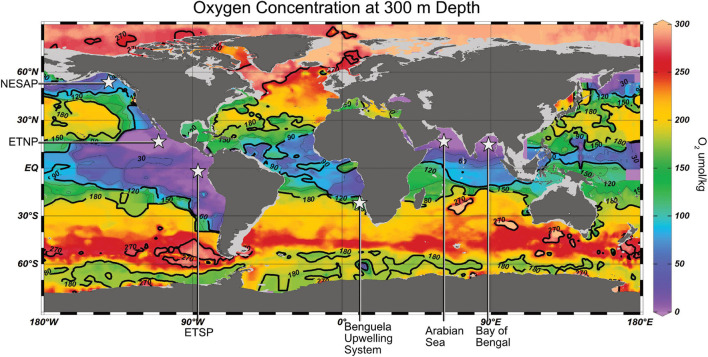
Global map of oxygen concentrations at 300 m depth. Data from NOAA World Ocean Database using all O_2_ data from 2009–2018. Data was plotted using Ocean Data View.

Oxygen minimum zones, using their simplest definition, exist where O_2_ is at its minimum in the water column and therefore occur globally. However, the magnitude and thickness of OMZs varies considerably; from oxic to hypoxic in much of the open ocean to functionally zero oxygen in anoxic marine zones (AMZs) such as the Eastern Tropical North and South Pacific (ETNP and ETSP) and the Arabian Sea (Figure 1). In addition to open ocean OMZs and AMZs, there are so-called “low oxygen” OMZs like the northeastern subarctic Pacific (NESAP), anoxic OMZs with sulfidic bottom waters such as the Cariaco Basin and Saanich Inlet, and seasonal hypoxic areas, which occur in many coastal environments like the Gulf of Mexico or in other oceanic regions such as the Baltic Sea. The various types of OMZs have different specific circumstances that lead to their formation but, in general, OMZs are engendered by physiochemical and biological processes such as thermal stratification, poor circulation, and eutrophy that drives biological productivity and oxygen consumption (Kessler, 2006; Karstensen et al., 2008; Paulmier and Ruiz-Pino, 2009). In addition to decreasing the solubility of O_2_ in seawater, rising temperatures are expected to contribute to all of these common factors in the creation of OMZs, which provide the basis of our understanding for the further expansion of OMZs (Oschlies et al., 2018).

Oxygen minimum zones have wide ranging effects on the ecology of organisms from every trophic level, which include range expansion or contraction and alterations in metabolic function. The availability of oxygen is one of the key factors that structures community composition and biogeographic range of marine organisms from bacteria and zooplankton to fishes and squids. While not the focus of this review, there are important effects on nekton that merit some discussion (reviewed in more detail here: Gilly et al., 2013). Recent results from hypoxic event models suggest that deoxygenation negatively affects crustaceans, fish, and mollusks even more than higher temperature or acidification through decreased survival, abundance, and metabolic functions (Sampaio et al., 2021). However, unless the deoxygenation drives local extinction of prey species, some predators might benefit; such as Humboldt squid, whose range has increased with OMZ expansion (Stewart et al., 2014), and elephant seals, which have been observed foraging in OMZs where a prey species, the ragfish, did not respond to the threat of predation (Naito et al., 2017). Likewise, deoxygenation is likely to benefit microbes with anaerobic metabolisms while possibly limiting the range for obligate aerobes. For instance, anaerobic microbes in OMZs greatly contribute to global biogeochemical cycles through N cycling in particular with up to 50% of the ocean’s N-removal occurring in OMZs (Codispoti et al., 1986; Bertagnolli and Stewart, 2018) and produce greenhouse gases [reviewed in Wright et al. (2012)]. As such, OMZs may contribute to climate change feedback loops where their expansion causes further greenhouse emissions and subsequent warming, which would then contribute to continued OMZ expansion.

A major goal of this review is to synthesize the current knowledge of OMZ formation, expansion, and the ecologies of viruses, bacteria, and microeukaryotes in these biogeochemically active systems. Our approach, which differs from other recent OMZ reviews (e.g., Ulloa et al., 2012; Wright et al., 2012; Bertagnolli and Stewart, 2018; Jurgens and Taylor, 2018; Wakeham, 2020), is to take into account all microbes, not just bacteria, and the underlying sediments of OMZs in addition to the water column. We then use this holistic description of OMZs in order to predict how marine deoxygenation might alter microbial communities and their processes. As this is inherently speculative in nature, we will conclude by identifying the current gaps in our understanding and make suggestions for how our field might address the vital question of how climate change driven deoxygenation will alter the base of marine ecosystems.

### Drivers of Oxygen Minimum Zone Formation

#### Abiotic Factors

The ocean is ventilated at the air-sea interface, which supplies oxygen to the surface mixed layer that is then transported to the global deep ocean at high latitudes (Luyten et al., 1983; Khatiwala et al., 2012). Oxygenated waters are also transported within basins by lateral advection mediated primarily by mesoscale eddies (Lee et al., 1997; Gnanadesikan et al., 2013; MacGilchrist et al., 2017; Bahl and Gnanadesikan, 2019). OMZs are often formed at midwater depths due to little lateral advection at these depths and the depletion of surface dissolved oxygen (Karstensen et al., 2008). These OMZs comprise ∼7–10% of oceanic volume and ∼1/3 of oceanic area (Paulmier and Ruiz-Pino, 2009; Bianchi et al., 2012).

In areas with low local ventilation, low lateral transport, and little basin-scale wind-driven circulation (Chavez and Messié, 2009), such as the ETNP (Busecke et al., 2019), ETSP (Paulmier and Ruiz-Pino, 2009), Arabian Sea (Resplandy et al., 2012), Benguela upwelling system (Schmidt and Eggert, 2016), and Cariaco Basin (Scranton et al., 1987), the OMZ can become functionally anoxic (Thamdrup et al., 2012). These OMZs are further exacerbated by upwelling of nutrient-rich waters to the surface, which increase biological productivity and therefore oxygen utilization at midwater depths (Busecke et al., 2019). However, some OMZs defined by riverine input such as the Bay of Bengal are less strong, likely due to the increased remineralization depth of their mineral-rich particles (Al Azhar et al., 2017).

Further, the size and strength of OMZs can follow seasonal patterns. While increased turbulence and lower temperatures in winter increase local ventilation (Zhang et al., 2018), most large OMZs such as the ETNP and ETSP do not exhibit significant seasonality (Paulmier and Ruiz-Pino, 2009). The Arabian Sea and Bay of Bengal, which are strongly and hydrodynamically influenced by monsoons, do not exhibit large seasonal trends in oxygen concentration despite vast seasonal differences in biological productivity (Resplandy et al., 2012; McCreary et al., 2013). Saanich Inlet, a seasonally anoxic fjord, experiences a strong OMZ during spring and summer due to low vertical mixing, but returns to oxic conditions in the winter due to an influx of oxygenated water from the NESAP (Herlinveaux, 1962; Lilley et al., 1982; Torres-Beltrán et al., 2017). Additionally, areas of coastal eutrophication, like the Gulf of Mexico hypoxic area, display strong seasonal trends due to excess nutrient input and increased summer stratification (Rabalais et al., 2002). Many abiotic factors contribute to the formation of OMZs and are inexorably linked to biotic factors as they allow for conditions that are amenable to further deoxygenation.

#### Biotic Factors

The main biotic input of dissolved oxygen into the ocean is photosynthetic primary production in the euphotic zone. Oxygen is primarily depleted *via* respiration, and when this rate exceeds the rate of oxygen input *via* biotic and abiotic factors, oxygen minimum zones can form (Rabalais et al., 2010). While OMZs are present throughout the ocean, dissolved oxygen can be completely depleted in areas with high productivity (Bianchi et al., 2012). OMZs are dominated by microbes (Wright et al., 2012; Bertagnolli and Stewart, 2018), as most multicellular organisms escape the low-oxygen area or are negatively impacted (Wishner et al., 2013). In upwelling regions such as the ETNP, ETSP, Arabian Sea, and Benguela upwelling system, microbial activity can be particularly high due to excess nutrients (e.g., Kalvelage et al., 2015). In the lower part of the euphotic zone of these strong OMZs, oxygen production *via* photosynthesis and utilization *via* respiration can be almost equivalent, resulting in a cryptic oxygen cycle (Garcia-Robledo et al., 2017).

Seasonal trends in coastal OMZ formation seem to be largely driven by biological productivity. During spring and summer, microbial blooms are more frequent and severe, directly depleting surface oxygen and increasing particle export to midwater depths that are broken down by diverse microorganisms (Wright et al., 2012). This can lead to more extreme OMZs than under fall and winter conditions. The biotic factors in OMZ formation and seasonality may be further enhanced by anthropogenic factors.

#### Anthropogenic Factors

Perhaps the most well-documented example of anthropogenic impacts on oxygen minimum zone formation is eutrophication-induced hypoxia, which affects many large fisheries including the Baltic Sea and northern Gulf of Mexico (Rabalais et al., 2001, 2002; Conley et al., 2002; Zillén et al., 2008). This eutrophication-induced hypoxia has affected over 500 sites globally (Diaz and Rosenberg, 2008) since the introduction of man-made nitrogenous fertilizers in the 1940s (Galloway et al., 2008). Most of these areas experience hypoxia in the spring and summer after phytoplankton blooms when stratification is strong, which often results in a decline of benthic organisms in coastal regions (Diaz and Rosenberg, 2008). Additionally, anthropogenic climate change has raised global temperatures, decreasing the solubility of dissolved oxygen into the surface ocean and increasing stratification (Stramma et al., 2008; Rabalais et al., 2010). Stratification increases the chance of deoxygenation as it prevents the equilibrium of subsurface ocean depths with the atmosphere due to the differences in density under the mixed layer. Anthropogenic, abiotic, and biotic factors all contribute to the predicted expansion and strengthening of OMZs.

### Expansion of Oxygen Minimum Zones Over Time

The expansion of low-oxygen areas has been well documented since the 1960s (Diaz and Rosenberg, 2008; Stramma et al., 2008; Keeling et al., 2010; Schmidtko et al., 2017; Breitburg et al., 2018). One major cause of this expansion is increased global temperatures, which decrease oxygen solubility and increase stratification (Keeling et al., 2010; Rabalais et al., 2010; Helm et al., 2011). Generally, increased temperatures also boost microbial productivity, further decreasing ocean dissolved oxygen as it is depleted through cellular respiration. These warmer temperatures may also strengthen winds that influence upwelling (Feely et al., 2008; Sydeman et al., 2014), resulting in yet more nutrients available at the surface to support biological production. As discussed above, increased nutrient loading from anthropogenic sources has contributed to the expansion of coastal hypoxia as well (Rabalais et al., 2010). However, the change in dissolved oxygen concentrations varies based on local hydrogeomorphology. For example, warming along the Chilean coast results in increased local ventilation and increased dissolved oxygen concentrations (Gnanadesikan et al., 2012). Even so, current climate models do not accurately replicate the observed deoxygenation in the global oceans, so there remains uncertainty regarding the exact causes of OMZ expansion (Oschlies et al., 2018). The continued deoxygenation of the world’s oceans will undoubtedly have an influence on the microbial ecology of OMZs, thus the current state of microbes in these environments must be addressed.

### Microbial Ecology of Oxygen Minimum Zones

The microbial community and activity within OMZs are fundamentally different compared to oxygenated waters and sediments. These differences are especially pronounced in the fully anoxic waters of AMZs, where anaerobic metabolisms thrive and aerobic metabolisms may only exist in microaerobic patches, sometimes caused by oxygen-generating processes such as photosynthesis (Tiano et al., 2014; Kalvelage et al., 2015) and possibly NO_2_^–^-dependent anaerobic methane oxidation (n-damo, Raghoebarsing et al., 2006; Ettwig et al., 2009). Further, microeukaryotes such as those belonging to Protozoa are known to be distributed along oxygen gradients from fully oxic to nearly anoxic (Fenchel, 2014). Likewise, viruses have unique communities in OMZs compared to over- and underlying oxic waters (Cassman et al., 2012; Mara et al., 2020; Vik et al., 2020). Thus, physiological and community composition differences are important in assessing how future deoxygenation events will expand or contract microbial niches within OMZs. As such, we will provide an overview of the diversity and physiological potential of bacteria, microeukaryotes, and viruses in the following sections before using these observations to speculate on how future deoxygenation events will alter the *status quo*.

### Prokaryotes

As in every marine environment, prokaryotes are the base of food webs in OMZs in both oxic and anoxic waters. In addition to contributing to carbon transfer through trophic levels in OMZs, prokaryotes are responsible for key processes in many biogeochemical cycles that have global implications. For instance, up to 35–50% of world-wide nitrogen loss occurs within the borders of OMZs (Devol et al., 2006), despite OMZs taking up only ∼7–10% of oceanic volume (Paulmier and Ruiz-Pino, 2009; Bianchi et al., 2012). Organisms within OMZs also contribute to sulfur (Callbeck et al., 2021) and methane (Chronopoulou et al., 2017) cycles, further cementing the importance of OMZs to global biogeochemistry. Community composition and genetic potential both inform how microbes can contribute to these processes; thus, we will first consider the current knowledge of OMZ community composition and how it differs between oxic, hypoxic, and anoxic regions within the water column.

Bacterial community composition is perhaps the most well-studied area of modern microbial ecology. Despite this, there are myriad fundamental questions that remain unanswered and there is some debate on which methodologies are most efficacious (e.g., Brumfield et al., 2020). Bacterial community composition has typically been assessed using one of three approaches: metagenomics, amplicon sequencing of 16S, or amplicon sequencing of functional genes. In general, studies using metagenomic or 16S sequencing seek to represent the entire bacterial community, while those utilizing functional gene or targeted 16S sequencing are designed to capture the diversity of bacteria that occupy specific niches within the whole community. A fully realized discussion of the benefits and caveats of these approaches is beyond the scope of this review and have been reviewed elsewhere (e.g., metagenomics: Nayfach and Pollard, 2016; 16S: Hugerth and Andersson, 2017), but a short summary of some of the key points is merited here as all OMZ community composition studies rely heavily on these methods. Although long considered the “gold standard” of bacterial community composition due to its assumed lack of primer bias, metagenomic sequencing is not without some caveats. For instance, metagenomic sequencing often requires steps that may cause highly abundant organisms or organisms with large genomes to be overrepresented. As previously mentioned, one of the primary drawbacks to 16S amplicon sequencing lies in primer bias, which can artificially inflate the relative abundance of specific taxa while depressing that of other taxa (Parada et al., 2016; Bukin et al., 2019). Even with these caveats, metagenomic and so-called “universal” 16S amplicon sequencing can provide an accurate representation of the total microbial community (Parada et al., 2016) and the improvement of the bioinformatic analysis of metagenomic and 16S sequences remain active areas of research (Straub et al., 2020). Finally, the analysis of functional genes from microbes (or targeted 16S gene primers) with specific niches in biogeochemical cycles have many of the same limitations as “universal” 16S gene analyses, but they provide an increased resolution of those organisms. However, functional gene and targeted 16S analyses cannot resolve the relative abundance of their targeted organisms compared to other bacteria present. Altogether, the three commonly used sequencing tactics provide complimentary information on the bacterial community and their genetic potential and studies employing them will thus be discussed in the following paragraphs.

Oxygen minimum zones are perhaps the ideal proving ground for the old adage that “Everything is everywhere, but the environment selects” (Becking, 1934). Differences in density along the water column prevent the mixed layer from reaching the seafloor in all but the most near-shore regions, providing a stratified system whose primary colonization routes are either from sinking particles from the overlying water column or currents from adjacent areas. Bacterial communities from adjacent areas can be excluded from this thought experiment as they will have similar hydrographic characteristics and thus similar selective pressures. Thus, despite being physically close, different depths in the same water column will have different selective pressures, which besides light availability perhaps none are stronger than changes in oxygen concentration and will therefore be expected to have quite different microbial communities. Walsh et al. (2016) explicitly tested this idea by using 16S sequencing across multiple depths from the surface to the sediment in three different regions in the Pacific Ocean: the ETSP, the open-ocean central equatorial Pacific, and the North Pacific gyre. In all three of these sampling sites, the OMZ contained unique communities compared to the other sampling depths. This trend of differing bacterial communities within OMZs compared to more oxygenated depths has been observed in other sampling sites within the ETSP (Beman and Carolan, 2013), as well as other OMZs such as the ETNP (Faull et al., 2020), the Bay of Bengal (Rajpathak et al., 2018; Fernandes et al., 2019, 2020; Lincy and Manohar, 2020), the Arabian Sea (Bandekar et al., 2018a; Fernandes et al., 2020), the Cariaco Basin (Zinger et al., 2011), and the Black Sea (Zinger et al., 2011). Taken all together, these findings suggest that dispersal may only add to species richness in OMZs when the species in question can persist in lowered oxygen conditions. While seemingly inconsistent with Baas Becking’s tenant, which is an obvious simplification, due to everything not actually being everywhere, a slight modification to “Everything *has the ability to be* everywhere, but the environment selects” allows the differences in community composition between OMZ and more oxygenated areas of the water column to fit within its framework.

While the methodologies used in these studies are not able to be directly compared, generalities about the community composition of prokaryotes from the surface to the OMZs can be made. Like in any oceanic region, Prochlorococcus, Marine Group II Euryarchaeota, SAR86, Verrucomicrobiales, Cellvibrionales, Actinomarina, Rhodobacterales, and SAR11 surface clades dominate the photic zone of many OMZs, even where the photic zones overlap suboxic and anoxic depths (e.g., Zaikova et al., 2010; Beman and Carolan, 2013; Bandekar et al., 2018a; Beman et al., 2020; Pajares et al., 2020). In contrast to oxic depths, OMZ prokaryotic communities often have higher relative abundances of Nitrospina, SAR202, SAR324, SAR406, Thaumarchaeota, Nanoarchaeota, and SAR11 deep clades in general (e.g., Zaikova et al., 2010; Beman and Carolan, 2013; Bandekar et al., 2018a; Beman et al., 2020; Pajares et al., 2020) and SUP-05 and Desulfobacteraceae in euxinic waters such as the Cariaco Basin (e.g., Rodriguez-Mora et al., 2015) and Saanich Inlet (Zaikova et al., 2010; Walsh and Hallam, 2011; Torres-Beltrán et al., 2019). While OMZ communities are likely to have endemic strains or amplicon sequence variants, the methodologies utilized to characterize prokaryote communities are too varied to make any concrete statements about the differences in community composition from one OMZ to another. As many studies on OMZ community composition speculate, the unique bacterial communities in OMZs are likely to reflect the redox conditions of OMZs compared to more oxygenated depths. The redox conditions of OMZs are often favorable for processes in the nitrogen cycle such as denitrification and anammox as well as other important processes in the sulfur and methane cycles. These redox conditions are reflected in the organisms present in OMZs such as known nitrogen and sulfur cycling organisms like genera belonging to *Candidatus* Scalindua, Caulobacteriaceae, Pelagibacteriaceae, α-Proteobacteria, δ-Proteobacteria, and γ-Proteobacteria in the Arabian Sea and Bay of Bengal (Bandekar et al., 2018a,b; Rajpathak et al., 2018; Fernandes et al., 2019, 2020; Amberkar et al., 2021), the Gulf of Alaska (Muck et al., 2019), Cariaco Basin (Madrid et al., 2001; Lin et al., 2008; Rodriguez-Mora et al., 2013, 2015; Cernadas-Martín et al., 2017), the ETSP (Stevens and Ulloa, 2008; Bryant et al., 2012), the ETNP (Podlaska et al., 2012; Beman and Carolan, 2013; Beman et al., 2020; Pajares et al., 2020), and Saanich Inlet (Zaikova et al., 2010; Walsh and Hallam, 2011; Torres-Beltrán et al., 2019). The contribution of prokaryotes identified in OMZs to biogeochemical cycles has global ramifications and has thus been well-studied throughout previous decades and remains a highly active research endeavor.

### Prokaryotic-Driven Nutrient Cycling

Much of what we know about microbe-driven nutrient cycling in marine environments stems from incubation experiments with labeled substrates, which, as was the case for sequencing, have several caveats that should be discussed before detailing their findings. Several reviews contain in-depth discussions on the considerations needed for individual methods for estimating rates in the nitrogen (e.g., Seitzinger et al., 1993; Zhang et al., 2020), sulfur (e.g., Jørgensen et al., 2019), and methane (e.g., Schulz, 2000) cycles. In general, all methods for determining rates of biogeochemical processes require careful sample collection schemes in order to obtain a representative sample in heterogeneous substrates and if the method requires bottle incubations, shorter incubation times are often required to reduce bottle effects and prevent the selection of microbes that favor these conditions. As such, many of the rates obtained are considered to be potential rather than actual rates and thus require careful interpretation.

**FIGURE 2:**
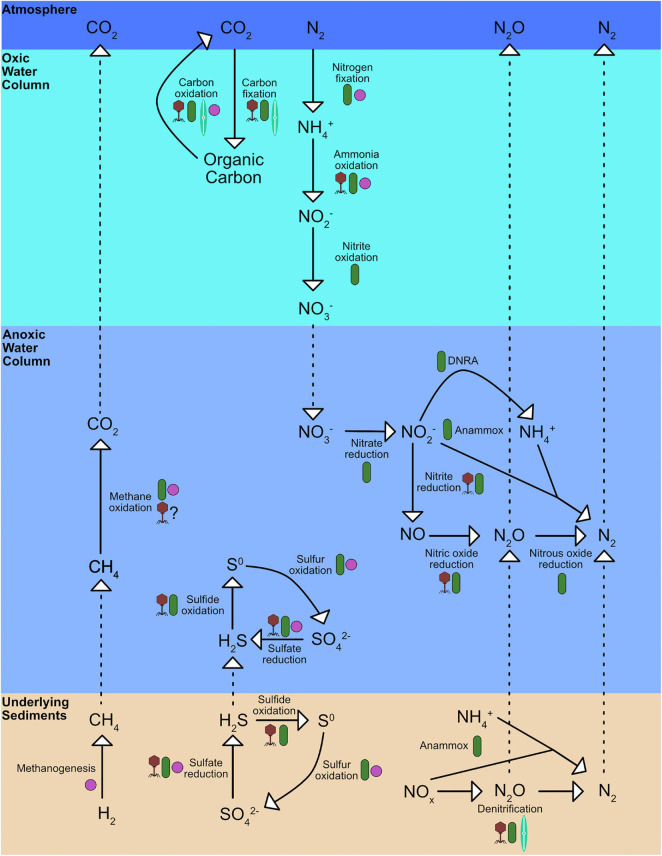
Simplified schematic of carbon, nitrogen, sulfur cycles in oxygen minimum zones, underlying sediments and overlying oxic waters. Purple cocci shapes represent processes that archaea perform, green rods represent bacteria, teal pennate diatoms represent microeukaryotes, and maroon phage particles represent processes viruses potential augment. Full details in text.

Prokaryotes are key contributors to nutrient cycles in OMZs and in over and underlying oxic depths within the water column. While there are anoxic microenvironments within oxic waters and oxic microenvironments within deoxygenated waters, the presence of oxygen, in addition to light availability and nutrient concentrations, is one of the key environmental factors that controls microbial metabolisms. The switch from heterotrophic consumption of carbon *via* respiration to fermentation is perhaps the most obvious difference, but the metabolic differences cascade through other nutrient cycles. For instance, the microbial-driven nitrogen cycle is typically separated between oxic and anoxic processes (Figure 2). Nitrification, the stepwise oxidation of NH_4_^+^ to NO_2_^–^ to NO_3_^–^, typically occurs in oxic environments. Anammox, the anaerobic oxidation of NH_4_^+^ with NO_2_^–^ to N_2_, denitrification, the stepwise reduction of NO_3_^–^ to N_2_O and N_2_, and DNRA, the dissimilatory reduction of NH_4_^+^ to NO_2_^–^, all usually occur in anoxic environments. Nitrogen fixation, the transformation of N_2_ to bioavailable N-species, has been observed in both oxic and anoxic environments.

Nitrification is carried out by archaea and bacteria in either an incomplete fashion with ammonium-oxidizing bacteria or archaea performing the first step of nitrification (NH_4_^+^ to NO_2_^–^) and nitrite-oxidizing bacteria completing the second step (NO_2_^–^ to NO_3_^–^) or in a complete fashion with “comammox” bacteria. Nitrifiers have been observed in the oxygenated waters overlaying OMZs (e.g., Stewart et al., 2012; Peng et al., 2013) and several studies have found copies of amoA, the gene encoding ammonium oxidase, present in anoxic waters (Peng et al., 2013). Furthermore, while nitrification activity is highest in oxic waters nearer the surface (e.g., Füssel et al., 2012; Bristow et al., 2016; Peng et al., 2016), several studies have observed NO_2_^–^ oxidation in anoxic depths (e.g., Lipschultz et al., 1990; Beman et al., 2013; Kalvelage et al., 2013; Peng et al., 2015). NO_2_^–^ oxidation in the absence of O_2_ requires an alternative electron acceptor, but this electron acceptor is currently unknown (Peng et al., 2016). Likewise, nitrogen fixation has been observed in both oxic and anoxic depths with higher rates of nitrogen fixation often occurring in the oxic waters nearer the surface (e.g., Fernandez et al., 2011, 2015; Jayakumar et al., 2017; Chang et al., 2019; Löscher et al., 2020). Nitrogen fixation is most commonly performed by cyanobacteria, including *Trichodesmium* (Capone et al., 2005) and UCYN-A (Zehr, 2011; Krupke et al., 2015), but heterotrophic diazotrophs belonging to α-Proteobacteria and γ-Proteobacteria also occur in many OMZs (Jayakumar and Ward, 2020).

Denitrification and anammox are the dominant N-loss processes in OMZs. Denitrification is carried out by myriad different genera including Pelagibacter (Tsementzi et al., 2016) and organisms belonging to α-Proteobacteria, δ-Proteobacteria, and γ-Proteobacteria in OMZs (e.g., Stevens and Ulloa, 2008; Jayakumar et al., 2009; Bandekar et al., 2018b). In addition to bacteria, a genome of potential archaeal denitrifier belonging to Marine Group II was recently assembled from metagenomic samples from the ETSP, which suggests that archaea may contribute to denitrification in OMZs (Sun and Ward, 2021). The genera of anammox bacteria found in most marine OMZs is *Candidatus* Scalindua (Woebken et al., 2007, 2008; Pitcher et al., 2011; Villanueva et al., 2014), while *Candidatus* Brocadia was recently found to be highly abundant in the Gulf of Alaska OMZ (Muck et al., 2019). The relative importance of denitrification and anammox toward the total N-loss seems to be dependent on regional and depth-related factors, with denitrification exceeding anammox in the Arabian Sea (Ward et al., 2009; Bulow et al., 2010), Saanich Inlet (Michiels et al., 2019) and in the OMZ core of the ETNP and ETSP but not in shallower depths still within the OMZ, where anammox contributed up to 80% of the N-loss (Babbin et al., 2020). Additional studies have found anammox to be the dominant N-loss process in the Benguela upwelling system (Kuypers et al., 2005), and in the ETSP in one study (Lam et al., 2009) but not in another (Dalsgaard et al., 2012). These discrepancies might be partially due to the influence of particles. The presence of particles is known to increase nitrite reduction, denitrification, anammox, and the encoding of particular genes involved in these processes (Ganesh et al., 2015). Further, anammox rates are influenced by the presence of small sinking particles such that anammox bacteria encounter appreciably more NH_4_^+^ when near particles (Karthäuser et al., 2021). While the relative contribution of anammox and denitrification may seem trivial to the overall picture at first glance, it becomes more important due to the fact that the denitrification pathway oftens stops at N_2_O in OMZs (e.g., Farías et al., 2009; Ji et al., 2015), thus producing a potent greenhouse gas. Additionally, N-loss processes can be sensitive to oxygen, as anammox and denitrification rates and the transcription of the genes involved decreased at nanomolar oxygen concentrations in the ETSP (Dalsgaard et al., 2014).

Oxygen minimum zones also provide a potential niche for prokaryotes that undergo dissimilatory reduction of nitrite to ammonium (DNRA). This process has been observed in several OMZs and may provide anammox bacteria with an additional source of NH_4_^+^ (Lam et al., 2009; Jensen et al., 2011). However, DNRA is sometimes undetectable in the ETSP (De Brabandere et al., 2014) and thus its importance in the nitrogen cycle may be limited. Despite this, prokaryotes with the genetic potential for DNRA are widespread across several OMZs, including the Arabian Sea (Lüke et al., 2016), the Alfonso Basin in the Gulf of California (Ramos-de la Cruz et al., 2021), and the ETNP (Pajares et al., 2019). Additionally, bacteria involved in n-damo, NO_2_^–^-dependent anaerobic methane oxidation, occur in OMZs and potentially link carbon and nitrogen cycling (Padilla et al., 2016; Chronopoulou et al., 2017). The n-damo process is carried out by bacteria from the NC-10 phylum (Raghoebarsing et al., 2006; Ettwig et al., 2009). The n-damo process produces N_2_ through the oxidation of CH_4_ *via* the reduction of NO_2_^–^ to NO, which is dismutated into N_2_ and O_2_. This newly produced O_2_ is then used as an oxidant for intra-aerobic methanotrophy. While NC-10 bacteria are present in several OMZs, their and n-damo’s importance in nitrogen and methane cycling has yet to be fully investigated. Despite this, they are transcriptionally active in the OMZ core of the ETNP, where it has been suggested that anaerobic CH_4_ oxidation is a substantial CH_4_ sink (Thamdrup et al., 2019). OMZs also support other methanotrophs due to the high concentration of CH_4_ within OMZ boundaries due to methanogenesis in the underlying bethos (Naqvi et al., 2010; Pack et al., 2015; Chronopoulou et al., 2017). While many of these methanotrophs have been identified in aerobic depths, some are also found within the OMZ core (Tavormina et al., 2013). As these methanotrophs require oxygen, they seem to partition based on oxygen concentration and may be associated with photosynthetic activity in the upper OMZ core (Torres-Beltrán et al., 2016; Padilla et al., 2017).

Sulfur metabolisms are another important nutrient cycle that occurs within the boundaries of OMZs. Callbeck et al. (2021) and van Vliet et al. (2021) have recently reviewed sulfur cycling in OMZs in detail beyond the scope of the present review, including describing key players and their metabolisms. Sulfur cycling in OMZ core waters has been elucidated with the detection of “cryptic” cycles in which sulfides are produced and utilized at nearly the same rate, thus resulting in sulfides at or below the detection limit (Canfield et al., 2010; Johnston et al., 2014; Carolan et al., 2015). Like methane, much of sulfur cycling in OMZs is directly linked to benthic nutrient cycling. Heterotrophic sulfate-reducing bacteria in sediments underlying OMZs are stimulated by inputs of organic matter (Fossing, 1990; Ferdelman et al., 1997, 1999; Brüchert et al., 2003). If the waters overlying these sediments remain stagnant for extended periods of time, sulfidic events may occur where benthically-produced sulfide reaches the water column which results in O_2_ and NO_3_^–^ consumption (Dale et al., 2016; Sommer et al., 2016). SUP05 bacteria, which display an extremely high affinity to sulfide (Crowe et al., 2018), are responsible for much of the sulfide-oxidation in OMZs after a sulfidic event and couple sulfide oxidation with the reduction of NO_3_^–^ (Callbeck et al., 2018, 2019). This clade often makes up a large proportion of the microbial community, as has been detected in the Benguela upwelling system (Lavik et al., 2009) and ETSP core OMZ waters (Callbeck et al., 2018). Even when sulfide concentrations are low, SUP05 bacteria have been found at high relative abundances due to mesoscale eddy-driven dispersal (Callbeck et al., 2018).

### Microeukaryotes

Microeukaryotes have, perhaps, an underappreciated role in OMZs. While comparably difficult to study in anoxic environments, microeukaryotes are important players in the carbon cycle, have the potential to exert top-down control upon prokaryotic communities *via* grazing, and some can even contribute to nutrient cycling *via* processes typically considered prokaryotic in nature such as denitrification. Even so, the community composition of OMZ microeukaryotes is the most well studied aspect of their ecology. Microeukaryotic community composition is typically assessed with 18S amplicon sequencing and is thus subject to the same caveats as 16S amplicon sequencing. In addition, 18S sequencing can be skewed by organisms that have many 18S gene copies (de Vargas et al., 2015). Metagenomic sequencing is rarely undertaken for microeukaryotes due to their large genome sizes but recovering eukaryotic genomes from metagenomes is becoming increasingly feasible with the development of new bioinformatic tools (e.g., Delmont and Eren, 2016; Krinos et al., 2021). The community composition of microeukaryotes in OMZs has been assessed in the Arabian Sea (More et al., 2018), the Black Sea (Wylezich et al., 2018), the Cariaco Basin (Edgcomb et al., 2011a,b; Orsi et al., 2011, 2012), the Costa Rica Dome (Jing et al., 2015), the ETNP (Duret et al., 2015), the ETSP (Parris et al., 2014; De la Iglesia et al., 2020), Framvaren Fjord (Behnke et al., 2006; Orsi et al., 2012), Saanich Inlet (Orsi et al., 2012; Torres-Beltrán et al., 2018), and Tolo Harbor (Rocke et al., 2016). One commonality between all of these studies is that the microeukaryotic communities in oxic waters were quite different from the communities in anoxic waters, which suggests that oxygen concentration has a profound effect on the presence or absence of certain microeukaryotic species. In these OMZs, the oxic waters, typically in the photic zone, were dominated by photosynthetic protists while the anoxic depths were dominated by putatively heterotrophic and parasitic protists. Beyond this trend, even heterotrophic Protozoa are likely to be distributed based on their oxygen tolerance and some are likely to be facultative anaerobes or facultative aerobes with the ability to persist in both oxic and anoxic habitats (Fenchel, 2014). Further, Rhizaria have also been found to partition vertically in the California Current ecosystem with the symbiont-bearing Acantharia and Collodaria near the surface, flux-feeding Phaeodaria in the lower epipelagic, and Foraminifera and other Phaeodaria populations near the OMZ (Biard and Ohman, 2020). Foraminifera have also been found in the OMZ core of the Arabian Sea (Gooday et al., 2000). The observations of Foraminifera near or in OMZs is of potential importance to the nitrogen cycle in OMZs as several benthic Foraminifera species are known to perform denitrification (Pina-Ochoa et al., 2010) and may contribute up to 50% of benthic denitrification in sediments underlying OMZs (Glud et al., 2009; Glock et al., 2013). Further studies of Foraminifera in the water column would be necessary to confirm their contribution to nutrient cycling in OMZs.

The potential for microeukaryotic grazing of prokaryotes is well established for oxic marine environments. The methods employed to estimate grazing rates in oxic waters are difficult to utilize in anoxic waters due to the likelihood of oxygen contamination from sampling with Niskin bottles (e.g., De Brabandere et al., 2012; Edgcomb et al., 2016) and thus *in situ* estimates may be better suited for OMZs (Medina et al., 2017). Even with these caveats, studies focusing on grazing found that up to 80% of prokaryotes were grazed daily in the Baltic Sea in anoxic waters (Detmer et al., 1993; Anderson et al., 2012), grazing in the OMZ of the Mediterranean Sea approached the level of grazing in the overlying oxic waters (Pachiadaki et al., 2016), grazing was negligible but selective in anoxic waters of the Cariaco Basin (Lin et al., 2007), and grazing in suboxic waters was higher than grazing in oxic waters of the ETSP (Cuevas et al., 2006). The inconsistency of results may be partially explained by the lack of *in situ* measurements of grazing or that the grazing pressure in separate OMZs can be drastically different. When using *in situ* methods, up to 28% of the total bacterial biomass may be consumed by protists in oxygen concentrations ∼4.8 μM and 13% of the biomass in completely anoxic waters in the ETSP (Medina et al., 2017). Taken all together, the loss of prokaryotes by grazing is an important piece of nutrient cycling in OMZs that is often overlooked.

### Viruses

Like the contribution of microeukaryotes, viruses in OMZs are understudied compared to prokaryotes in these environments. This is despite the recognized roles that viruses play in marine environments such as exerting top-down controls of prokaryotic and eukaryotic populations alike, modulating host metabolisms, and even transferring nutrients if they are consumed by organisms. For instance, the percent of microbial mortality due to viral lysis was estimated to be 2–50% in hypoxic depths in the Baltic Sea (Weinbauer et al., 2003; Anderson et al., 2012), with estimates generally higher in anoxic samples. High virus-induced prokaryotic mortality (up to 90%) has also been observed in an anoxic marine-influenced lake near the Adriatic Sea (Rastelli et al., 2016). Though one study in the subtropical western Pacific shows a relative switch from grazer-driven to virus-driven mortality with increasing depth (Tsai et al., 2016), other studies in the Mediterranean Sea and Arabian Sea estimated viral production and viral-induced prokaryotic mortality to decrease with depth (Weinbauer et al., 2003; Parvathi et al., 2018). Thus, the relationships between the contributions of viral lysis and grazing to prokaryotic mortality based on depth and oxygen concentration have not been resolved. Additionally, there is some evidence that OMZs may have a higher incidence of lysogeny than the surrounding oxygenated waters. Changes in nutrient availability, productivity, and oxygen concentration can cause stress to host organisms and subsequently cause viruses to enter the lysogeny cycle (Howard-Varona et al., 2017; Correa et al., 2021). Direct estimates of lysogeny *via* prophage induction in OMZs have been generated for the Arabian Sea (Parvathi et al., 2018) and Baltic Sea (Weinbauer et al., 2003). In both cases the percentage of lysogens was estimated to increase between the surface and low-oxygen waters, though the percent lysogeny decreased in the suboxic samples in the Baltic Sea and increased in the Arabian Sea. In both cases, oxygen contamination in incubations was difficult to prevent and not directly measured, potentially resulting in an altered response from the microbial community that may have affected virus-host interactions. Whether the aforementioned trends in virus-host interactions hold true for all OMZs or even within the same OMZs over multiple seasons remains an open question.

To date, the total virus community composition has only been assessed in the ETSP (Cassman et al., 2012; Vik et al., 2020), the Cariaco Basin (Mara et al., 2020), and Saanich Inlet (Chow et al., 2015) and the putative archaeal virus community has been assessed in the ETNP (Vik et al., 2017). In each OMZ, viral community composition was appreciably different in anoxic samples compared to oxic samples. In the case of the ETSP (Vik et al., 2020) and Cariaco Basin (Mara et al., 2020), some identified viral populations were detected in other OMZs contained within the Global Ocean Virome 2.0 dataset (Gregory et al., 2019), suggesting that some viral populations specialized for low-oxygen environments may be distributed across global OMZs. However, this represented a small fraction of the total virus populations which hints at a majority of virus populations in OMZs being endemic to their OMZ of origin. The question of cosmopolitan or endemic virus populations requires more OMZ viral communities to be sequenced and a direct comparison to be undertaken.

Additional challenges in understanding the roles of viruses in OMZs include the issues that viruses with RNA genomes are not included within the current studies, and that relatively few DNA viral populations from OMZ studies could be assigned a taxonomic identification. This latter issue is largely due to most viruses being known only from metagenomic sequencing data and lacking cultivated representatives in genomic databases. Thus, more endeavors to cultivate viruses from OMZs, and indeed all marine environments, must be undertaken to gain a more comprehensive understanding of the roles of viruses in these environments. This challenge of matching viral populations with host microbial populations is the biggest obstacles in understanding environmental viruses. As the labor involved in cultivating new viruses would take decades to fill in all the potential holes in our knowledge, a number of bioinformatic techniques are being actively developed to match viruses to hosts. These approaches either rely on *in silico* or *in vitro* methods. The *in silico* methods are varied and numerous, with nine published in the past 2 years alone [reviewed in Coclet and Roux (2021)] but all require databases of both host and virus sequences, ideally paired from the same study. The *in vitro* methods are also numerous [reviewed in Coclet and Roux (2021)] but as many of them rely on probes and PCR primers, their utility in OMZs is limited to viruses that are close relatives to each other. High-throughput chromosomal confirmation capture (Hi-C) has been used to link viral and host DNA (Bickhart et al., 2019; Marbouty et al., 2021), thus capturing a close relationship, indicting either infection, lysogeny, or attachment. Hi-C has been further adapted to link viral RNA with host DNA, with the idea being that this allows the user to capture an ongoing infection (Ignacio-Espinoza et al., 2020). Both the *in silico* and *in vitro* methods show great promise for elucidating virus-host pairings and should be undertaken in OMZ samples.

Viruses influence host metabolism during infection, not only through diverting substrate usage to building viral machinery, but also through possibly supplementing key components within host energetic pathways *via* viral auxiliary metabolic genes (AMGs). The most thoroughly studied and best understood AMG is *psbA*, which codes for a photosystem II protein, in bacteriophages that infect cyanobacteria. As we reach the 20th anniversary of this discovery (Mann et al., 2003), our understanding of AMGs has rapidly evolved. For instance, viral *psbA* expression can sometimes account for over half of all *psbA* expression, highlighting the potential of viruses to directly affect important host metabolisms and biogeochemical cycles (Sieradzki et al., 2019). However, the relative abundance of psbA genes in T4-like cyanophages decreases with depth in the ETNP, which may be reflective of its low-light adapted Prochlorococcus host in anoxic chlorophyll maxima and suggests other AMGs are more important in OMZs (Fuchsman et al., 2021). For instance, several putative AMGs have been identified from the Cariaco Basin, the ETNP, the ETSP, and Saanich Inlet (Roux et al., 2014; Ahlgren et al., 2019; Mara et al., 2020; Vik et al., 2020; Gazitúa et al., 2021). Some of the putative AMGs found within OMZs and their overlying oxic waters potentially contribute to important processes in the carbon, nitrogen, and sulfur cycles (Figure 2). For instance, viruses carrying *dsrC* genes, which are involved in dissimilatory sulfate reduction, were found in Saanich Inlet that putatively infect SUP05 bacteria (Roux et al., 2014). AMGs putatively involved in sulfite oxidation, *soxY*, have also been observed in the ETSP (Kieft et al., 2021). Additionally, viruses carrying putative AMGs from nitrogen cycle pathways have been observed in OMZs such as *amoC*, which encodes for ammonia monooxygenase subunit C involved in nitrification, *nirK*, which encodes for a Cu-containing nitrite reductase involved in denitrification, and *norB*, which encodes for a nitric oxide reductase involved in denitrification (Ahlgren et al., 2019; Gazitúa et al., 2021). Finally, viruses carrying the *pmoC* gene, a subunit of the particulate methane monooxygenase enzyme that catalyzes methane oxidation, have been observed in freshwater lakes (Chen et al., 2020) and soils (Lee et al., 2021). This indicates that viruses have the potential to augment methane oxidation by their methanotrophic hosts, even though they have yet to be observed in OMZs. While these carbon, nitrogen, and sulfur cycle AMGs have not been expressed in controllable systems to confirm their function, nor has their expression in nature been fully observed, the presence of these putative AMGs in virus genomes points at the vast potential of viruses to affect global nutrient cycles.

### Sediments

The sediments underlying OMZs can be important sources of carbon, nitrogen, and sulfur species that have the ability to influence the ecology of OMZ cores. In some OMZs, the anoxic waters may even reach the benthos. As such, a complete understanding of OMZs is impossible without taking sediments into consideration. Despite this, sediments are understudied compared with the overlying water column. As discussed above, sediment sulfur cycling can introduce sulfide into the overlying water column, partially due to the higher relative sulfate reduction in sediments where OMZ cores reach nearer the bottom waters (Fernandes et al., 2018). Methanogenesis is also known to occur in sediments where the OMZ meets the continental shelf, which contributes to the large methane reservoirs in OMZs (Pack et al., 2015; Chronopoulou et al., 2017; Thamdrup et al., 2019). Further, both sulfate reduction and methanogenesis have appreciably higher rates in sediments where the OMZ core reaches the benthos in the ETSP (Maltby et al., 2016). The role of sediments in nutrient cycling extends to the nitrogen cycle as nitrification, denitrification, anammox, and DNRA all occur in the ETSP, where the sediments contribute to a net loss of N where the bottom waters are anoxic or nearly anoxic (Bohlen et al., 2011). Denitrifiers are also found in the sediments underlying the Arabian Sea (Lincy and Manohar, 2020; Amberkar et al., 2021), the Bay of Bengal (Lincy and Manohar, 2020), and the ETNP (Liu et al., 2003). Even the sediments in less intense OMZs, such as the NESAP, can greatly contribute to N_2_O production from denitrification and is largely modulated by the oxygen concentration of bottom waters (Jameson et al., 2021). Likewise, the effect of low bottom water oxygen concentrations is felt by the prokaryotic communities as well, with different microbes occurring in sediments with oxic bottom waters compared to those with anoxic bottom waters (e.g., Divya et al., 2011; Divya, 2017; Besseling et al., 2018; Bhattacharya et al., 2020; Lincy and Manohar, 2020; Amberkar et al., 2021). Unfortunately, there have been no studies characterizing the community composition of viruses in sediment underlying marine OMZs. However, two studies in anoxic Mediterranean Sea sediments found viruses occurred in anoxic sediments at similar abundances as they did in nearby oxic sediments (Danovaro et al., 2005), and have much higher viral production rates in anoxic than in oxic sediments (Corinaldesi et al., 2014). While these studies were not explicitly in OMZs, this suggests that viruses may have strong effects on cellular life in sediments impacted by OMZs. Taken all together, the sediments underlying OMZs are both influenced by and influence the microbial ecology of the water column, with the most dramatic effects occurring in regions where the OMZ core reaches the sediment-water interface.

With the expansion of OMZs, we must also consider the potential impacts on the fauna residing on or near the seafloor. OMZs can impact the benthos through shifting community structures, as seen in ETSP meiofauna (Neira et al., 2018) and ETNP benthic decapod crustaceans (Papiol et al., 2017). Further, differences in oxygen concentration may alter coral community composition (e.g., Hanz et al., 2019; Hughes et al., 2020). For instance, the Angolan margin was able to support cold-water coral mounds, while the Namibian margin could not, possibly due to lower dissolved O_2_ concentration (Hanz et al., 2019). Changes in coral species distributions are likely to impact the distributions of their microbial symbionts and could shift their metabolisms (Hughes et al., 2020).

### Response of Microbes to Climate Change and Continued Deoxygenation

While this review focuses on the potential effects of climate change-driven deoxygenation on marine microbial ecology, other major climate alterations may help cause feedback loops that result in continual deoxygenation. For instance, warming is expected to increase prokaryotic respiration rates (e.g., Wang et al., 2021), which, in turn, has the potential to stimulate more and stronger deoxygenation events (Oschlies et al., 2018; Robinson, 2019). Acidification seems to have a negligible effect on prokaryotes overall (Wang et al., 2021), but several studies have found that nitrification is inhibited by acidification (Huesemann et al., 2002; Beman et al., 2011; Kitidis et al., 2011; Braeckman et al., 2014; Wannicke et al., 2018), which, while not directly related to OMZs, would likely have an effect on the availability of NO_3_^–^ and NO_2_^–^ for denitrifiers and anammox bacteria. The direct effect of increased temperature and lowered pH on OMZ microeukaryotes is less clear. Acidification will likely decrease the abundance of any calcium test-bearing microeukaryotes in OMZs like it does for coccolithophores (D’Amario et al., 2020). Further, rising temperatures have the potential to increase grazing rates of bacteria by microeukaryotes in oxic waters (Gu et al., 2020; Cabrerizo and Marañón, 2021), but it is unclear whether this can overcome the observed negative effect low oxygen concentrations have on grazing. As for viruses, warming has the potential to increase decay rates, especially in tropical regions (Danovaro et al., 2011), and acidification seems to have the ability to select for particular coccolithophore virus strains over others (Highfield et al., 2017) but whether this is a general trend for all viruses has not been ascertained.

**FIGURE 3:**
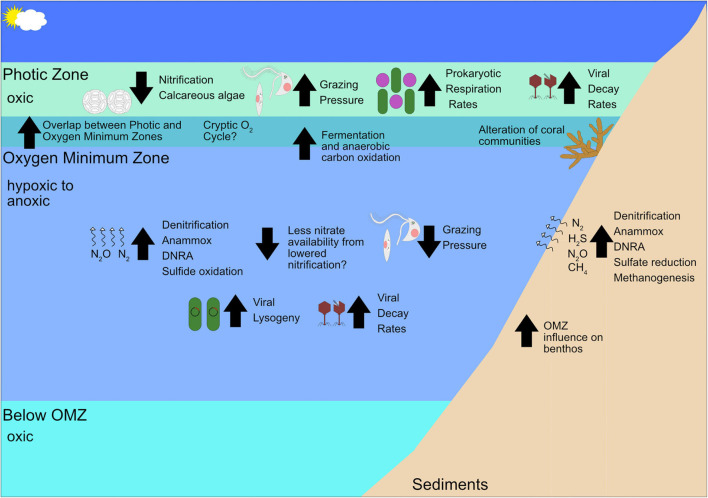
Schematic of how future deoxygenation may affect the ecology of OMZs and their adjacent areas. Upward pointing arrows indicate the process or organisms whose abundance/rate is expected to increase, not considering biotic interactions and feedback loops by climate change and downward pointing arrows indicate negative effects.

The continued deoxygenation of the ocean will result in thicker and more intense OMZs. Based on the differences between oxic and anoxic depths detailed in this review, deoxygenation has the ability to drastically change the microbial community in marine environments by shrinking the available niches of aerobic organisms and greatly expanding the range of anaerobes (Figure 3). These changes can potentially reverberate through trophic levels. Further work on how trophic linkages differ between oxic and anoxic waters is needed to gain a fuller picture of how food webs will be altered. Even so, it is likely that prokaryotic, microeukaryotic, and virus communities will shift to strains or species more tolerant of low oxygen conditions. This shift will, in turn, favor anaerobic metabolisms such as fermentation, denitrification, anammox, sulfate reduction, methanogenesis, etc. In the case of denitrification and methanogenesis, favorable conditions for anaerobic metabolisms may cause further greenhouse gases to be released from marine systems if the increase in these processes outstrips the utilization of N_2_O and CH_4_ by marine microbes. This is of particular importance when considering that regions with less intense OMZs like the Gulf of Mexico may act as N_2_O and CH_4_ sources rather than sinks (Rogener et al., 2021). Further, microeukaryotic grazing of prokaryotes may decrease and the abundance of parasitic microeukaryotes may increase. The overall virus community will shift to viruses that infect hosts utilizing anaerobic metabolisms and the percentage of viruses that undergo lysogeny may increase. Benthic communities are likely to change as well, with higher instances of anaerobic processes such as denitrification, anammox, sulfur oxidation, and methanogenesis and shifts in coral communities are likely to be altered by deoxygenation. Benthic macrofauna communities are also likely to be altered even in hypoxic areas like the Gulf of Mexico, where regions experiencing hypoxia are more likely to provide habitats for polychaetes rather than the bivalves, pericaridean crustaceans, gastropods, and ophiuroids typically in oxygenated benthic environments (Rabalais et al., 2002). Further, viruses may have an increased importance in causing prokaryotic mortality with increasing deoxygenation as viral lysis has been observed to increase in suboxic relative to oxic waters (e.g., Anderson et al., 2012; Rastelli et al., 2016), but other studies show conflicting evidence (e.g., Weinbauer et al., 2003; Parvathi et al., 2018), so this potential effect is not reflected in Figure 3 due to the uncertain nature of virus-host responses to deoxygenation. As these predictions based on the literature are speculative in nature, we will close by offering some suggestions for how future research on marine microbes should be conducted to fill the gaps in our knowledge.

First, a concerted effort should be undertaken to study how climate change will alter marine microbes in a holistic manner. While extremely valuable information can be gleaned from studies targeting the response from one group of organisms, how linkages between microeukaryotes, prokaryotes, and viruses will be altered from climate change cannot be assessed without studies that track all of these biological entities. Secondly, there is a desperate need for more anoxic and/or *in situ* incubations to estimate important processes in OMZs such as grazing and viral lysis, lysogeny, and how these and other processes affect biogeochemical cycling. Even though prokaryotes are the best studied microorganisms in OMZs, there is a clear need for research examining the differences between communities in different OMZs globally and how they differ in transitional OMZs that are likely to have decreased oxygen concentration in the future. The need for more studies on microeukaryotes and viruses in OMZs is evident from the sparsity of the literature presented in our review and should be a main target for future research endeavors. Metatranscriptomics may be used to assess the activity of viruses as they infect cells within OMZs, including how the expression of the newly discovered nitrogen cycling putative AMGs compare to their host homologs. Even at a more basal level, the total virus community of OMZs should be assessed in seasonally hypoxic areas such as the Gulf of Mexico hypoxia and other coastal environments that will experience further deoxygenation from climate change.

## Author Contributions

AL and JB devised the structure and content of the review. AL primarily wrote the first draft with the assistance of SJ, AP, and ES. All authors edited the subsequent drafts of the manuscript.

## Conflict of Interest

The authors declare that the research was conducted in the absence of any commercial or financial relationships that could be construed as a potential conflict of interest.

## Publisher’s Note

All claims expressed in this article are solely those of the authors and do not necessarily represent those of their affiliated organizations, or those of the publisher, the editors and the reviewers. Any product that may be evaluated in this article, or claim that may be made by its manufacturer, is not guaranteed or endorsed by the publisher.
